# Leader of the SAC: molecular mechanisms of Mps1/TTK regulation in mitosis

**DOI:** 10.1098/rsob.180109

**Published:** 2018-08-15

**Authors:** Spyridon T. Pachis, Geert J. P. L. Kops

**Affiliations:** Oncode Institute, Hubrecht Institute – KNAW and University Medical Centre Utrecht, Utrecht, The Netherlands

**Keywords:** Mps1, TTK, spindle assembly checkpoint, kinetochore, aneuploidy, chromosome segregation

## Abstract

Discovered in 1991 in a screen for genes involved in spindle pole body duplication, the monopolar spindle 1 (Mps1) kinase has since claimed a central role in processes that ensure error-free chromosome segregation. As a result, Mps1 kinase activity has become an attractive candidate for pharmaceutical companies in the search for compounds that target essential cellular processes to eliminate, for example, tumour cells or pathogens. Research in recent decades has offered many insights into the molecular function of Mps1 and its regulation. In this review, we integrate the latest knowledge regarding the regulation of Mps1 activity and its spatio-temporal distribution, highlight gaps in our understanding of these processes and propose future research avenues to address them.

## 1. It's got to be perfect: faithful chromosome segregation by attachment error correction and the spindle assembly checkpoint

When a cell divides, the two resulting daughter cells each inherit an exact copy of its genetic content in order to maintain healthy cell function. Equal genome inheritance is driven by the mitotic spindle. Microtubules emanating from opposite poles of the spindle capture structures known as kinetochores on the two sister chromatids of chromosomes that were formed during the genome replication phase of the cell cycle. The sister chromatids separate only when every chromosome has achieved biorientation, a state in which one sister chromatid is attached to microtubules emanating from only one of the two poles while the other has attachments only to the other pole [1]. The result is the distribution of a complete copy of the genome towards opposite ends of the dividing cell, allowing fission to generate genetically identical daughters [2]. Compromised fidelity of chromosome segregation is implicated in a number of pathologies including cancer and in defects in embryonic development [3–6]. Given the approximately 3 × 10^13^ cell divisions needed to make a human [7] and the roughly 50 billion cell divisions that take place in the human body every day, the necessity for a surveillance and correction system that ensures faithful chromosome segregation becomes apparent. In eukaryotic organisms, this system is a coordinated effort of two processes: attachment error correction and the spindle assembly checkpoint (SAC). The SAC operates as a molecular surveillance mechanism that essentially monitors the attachment status of each kinetochore [8,9]. Unattached kinetochores generate SAC signalling by forming a tetrameric protein complex known as the mitotic checkpoint complex (MCC). MCC prevents chromosome segregation and mitotic exit by inhibiting the anaphase promoting complex (APC/C) [10–13]. When microtubules bind to kinetochores, SAC signalling from those kinetochores is switched off. Complete SAC silencing occurs once all kinetochores have achieved stable attachments, leading to termination of all MCC production, liberation of APC/C and initiation of anaphase [14,15]. The error-correction pathway is intimately connected to the SAC. For example, any wrongly attached chromosome will be detached from microtubules, resulting in reactivation of SAC signalling. In addition, the main orchestrator of the error-correction pathway, the Aurora B kinase, directly impacts SAC signalling in several ways, and a number of SAC components have important roles in error correction [16–27]. Central in the connection of the processes is Mps1/TTK (hereafter referred to as Mps1), an evolutionary conserved kinase that is indispensable for error correction, and that is the chief conductor of the SAC [28].

## 2. All about that kinase: some Mps1 basics

A few years after the initial discovery of budding yeast Mps1 [29] and its identification as a kinase [30], two human cDNA library screens for proteins recognized by anti-phospho-tyrosine antibodies identified a kinase (named PYT (phospho-tyrosine picked threonine kinase) and TTK, respectively) that had homology to yeast Mps1 [31,32]. PYT/TTK was recognized as a serine/threonine kinase but was designated as a dual-specificity kinase because of its ability to phosphorylate tyrosines *in vitro*. However, there is currently no evidence that functionally relevant phosphorylation events by Mps1 in cells occur on tyrosines. Mps1 does, however, have a strong *in vitro* preference for threonines over serines, and indeed, most Mps1 substrates are phosphorylated on threonines that lie in a PLK1-like E/D-x-T motif [33,34]. The initial experiments describing a function for Mps1 linked it to an M-phase checkpoint: budding yeast Mps1 mutants were defective in their ability to arrest in response to spindle poisons, while Mps1 overexpression caused spontaneous M-phase arrest [35,36].

**Figure 1.**
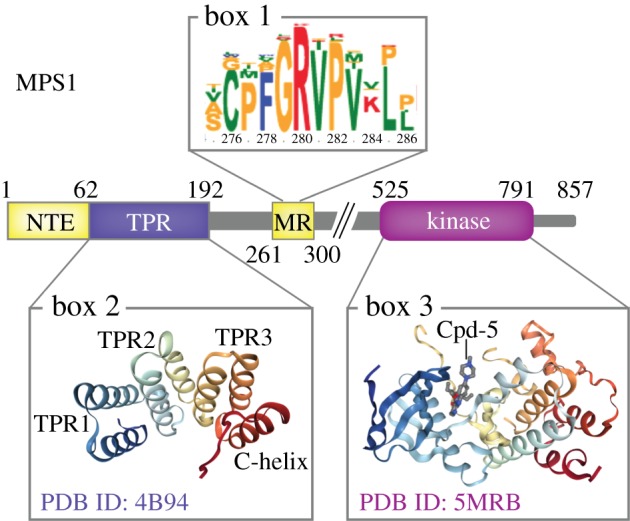
Domain organization and important features of human MPS1. The enzymatic domain is located near the C-terminus (box 3, depicted in complex with the small molecule inhibitor Cpd-5 to highlight the ATP-binding pocket). The other functionally characterized sequences are involved in MPS1 activation and kinetochore localization. The NTE and MR are both important for interactions with the NDC80 complex, but only the MR sequence is conserved in eukaryotes (box 1). The TPR domain (box 2) is involved in the regulation of MPS1 recruitment to kinetochores and has a structure similar to TPR domains of the SAC proteins BUB1 and BUBR1.

Mps1 orthologues can be identified in all supergroups of eukaryotes and in all metazoa, with the exception of nematodes [37]. All eukaryotic Mps1 orthologues have a similar C-terminal kinase domain (figure 1, box 3) [38], but differ in their N-terminal sequences. In contrast to most fungi, vertebrate Mps1 proteins harbour an N-terminal tetratricopeptide repeat (TPR) domain (figure 1, box 2), involved in regulating its subcellular localization to kinetochores and centrosomes. This domain is ancient but was lost in many eukaryotic lineages, for unknown reasons [39]. The stretch of protein sequence between the TPR domain and the kinase domain is substantial, but little is known about its relevance.

Though the SAC is essential in many but not all model organisms, Mps1's involvement in a number of additional processes (such as error correction) makes it essential for cell viability in many of them, including human cells [40–42]. It is expressed in the majority of proliferating tissues examined in humans (as found in a number of different studies [43,44] and the Human Protein Atlas), and its protein and kinase activity levels are cell cycle-dependent: they peak in early mitosis and decline rapidly as cells re-enter the G1 phase of the subsequent cell cycle [45,46]. Degradation of Mps1 at the end of mitosis is subject to regulation by the APC/C-Cdh1, effectively ensuring irreversible suppression of the SAC pathway upon G1 entry [47]. Mps1 levels are slowly regained during interphase, when it starts localizing to the nuclear envelope [46]. Despite low activity in interphase, it was reported that interphasic functions for Mps1 exist, including a role in the G2 DNA damage response [48–50] and in centrosome duplication [51–55]. These findings are somewhat controversial, and it will be important to show that acute inhibition of Mps1 during interphase impairs these processes.

## 3. Get busy: the orchestration of error correction and spindle assembly checkpoint by Mps1

Once activated in early mitosis [21], Mps1 starts an impressive multitasking feat, phosphorylating a number of substrates that simultaneously promote error correction and MCC production. Mps1 impacts on error correction by two main routes. Although not consistently seen in all conditions [51,56–58], Mps1 can regulate Aurora B activity and localization [21,24]. Mps1 additionally phosphorylates the Dam complex in yeast and the Ska complex in human cell lines. These complexes are analogous [59], and they promote attachment of kinetochores to dynamic microtubule plus-ends [60–63]. Mimicking the phosphorylation of the Ska complex in cells destabilizes kinetochore–microtubule attachments, but whether it contributes to human MPS1's role in error correction has not been directly tested. Given that a phospho-proteomics screen uncovered dozens of potential mitotic human MPS1 substrates, it may have yet more targets that impact microtubule attachments [62].

While Mps1 is busy ensuring error correction, it also initiates a cascade of events that lead to SAC activation. MCC production requires sequential recruitment of a number of SAC proteins to kinetochores eventually culminating in the local enrichment of all components of the MCC [64–67]. Mps1 phosphorylates multiple residues on at least three proteins involved in this recruitment cascade (Knl1, Bub1 and Mad1) [68,69] and may subsequently also directly impact on MCC complex stability and APC/C binding [70]. For the remainder of this review, we will focus on the events leading to Mps1 localization and activation, and we refer to several papers for more detailed insights into the SAC events downstream of Mps1 activity [67,68,71–73].

## 4. Start it up: molecular events leading to Mps1 activation

Mps1 activity peaks in mitosis, and this likely relies on *trans*-autophosphorylation on several residues in the activation loop of Mps1 or close to it (P + 1 loop) [74–77]. Chemically inducing Mps1 dimerization in cells is sufficient to activate the kinase [74]. Mps1 indeed dimerizes in cells, and this promotes autoactivation [56]. The mechanism by which Mps1 dimerizes is unknown, and studies have provided conflicting evidence as to whether the N-terminal region including the TPR domain is involved in this [39,78]. The N-terminal region known as the N-terminal extension (NTE, see below) does affect kinase activation in another way: by directly inhibiting the kinase domain [79]. In any case, dimerization, oligomerization or clustering facilitates activation of Mps1, possibly by releasing NTE-mediated inhibition of the kinase domain and certainly by facilitating *trans*-autophosphorylation of the activation loop in Mps1. It is likely, albeit not formally shown, that this all takes place at kinetochores, which are required for mitotic Mps1 function. However, the initial activation may also occur elsewhere. Mps1 has an important role in establishing a pool of interphasic MCC [51], which may be important for restraining the APC/C until unattached kinetochores become competent for assembling the bulk of MCC [1]. Assembly of interphase MCC complexes takes place on the nuclear pore complex (NPC) [80], where Mps1 and some other SAC components reside [46,81–83]. Mps1 is indeed imported into the nucleus prior to mitosis, possibly via a KKRGKK motif near its amino-terminus [31] or via two N-terminal import signals (although their position and orientation may be incompatible with import factor binding) [84]. It seems likely therefore that at least some nuclear Mps1 activity is generated before kinetochores are assembled but it is unknown if that activity comes from an NPC-localized pool or, for example, from a diffusible nucleoplasmic pool of Mps1. Such insight will have to await activity biosensors and mechanistic information on how Mps1 localizes to the NPC.

## 5. Come together: how Mps1 binds kinetochores

Once the nuclear envelope breaks down, Mps1 localizes to kinetochores, where its main substrates for error correction and the SAC reside. Initial studies mapped the kinetochore-binding region of human MPS1 to its N-terminal 300 amino acids [46,51,55,85–87]. Sequence and structural analysis of this region identified three tandem TPRs capped by a seventh helix [78] that together form a TPR domain (figure 1, box 2) [39] with high structural similarity to TPR domains of the SAC proteins Bub1 [88] and BubR1 [89–91]. Our preliminary comparative genomics efforts indeed suggest that the Mps1 and Bub1/BubR1 TPR domains have shared ancestry, resulting from a duplication before the last eukaryotic common ancestor (van Hooff and Tromer 2018, unpublished). The three tandem repeats form a concave C-shaped cross-section, which in other TPR-containing proteins are involved in ligand binding [39]. To this day, however, no ligands have been identified for the Mps1 TPR domain. Ligand binding may also occur on the convex side, as it does for the Bub1 and BubR1 TPR domain interactions with Knl1 [92,93]. The TPR domain in vertebrate Mps1 proteins is preceded by an approximately 60 amino acid NTE with unknown structural properties and limited sequence conservation in eukaryotes (figure 1) [39]. Both the TPR domain and the NTE possess an affinity for the kinetochore, but the NTE appears to provide the dominant localization potential [39,58]. It is unknown what molecular features of Mps1 regulate its kinetochore localization in non-vertebrates, but a study in budding yeast suggested that its N-terminal regions are important for kinetochore-dependent functions [86].

**Figure 2.**
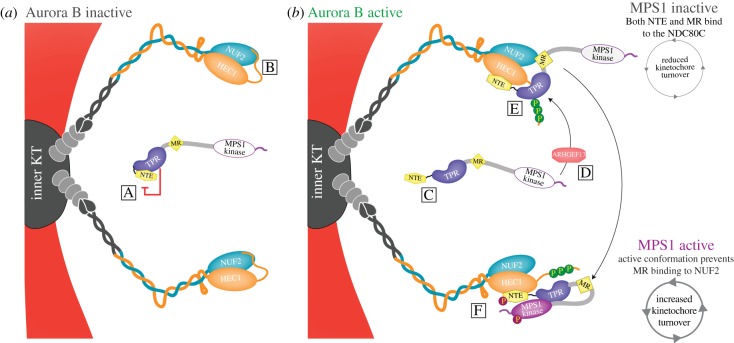
An integrated model of MPS1 interactions with the NDC80 complex and the regulation by Aurora B in humans. In the absence of Aurora B activity (*a*), the TPR domain inhibits the NTE (A) and the HEC1 tail interacts with NUF2 thereby blocking the MR–NUF2 interaction (B). When Aurora B is active (*b*), the NTE is released from TPR inhibition. ARHGEF17 interacts with inactive MPS1 (D) and brings it to the kinetochore where it binds to the NDC80 complex via both the NTE and MR regions (E). The phosphorylation of the tail of HEC1 by Aurora B frees up the binding site in NUF2 and allows the MR to bind there. Binding of inactive MPS1 to the NDC80C via both regions causes relatively tight binding and leads to relatively long kinetochore residence time. The slow turnover promotes MPS1 autoactivation which adopts a different conformation and is now able to bind only via the NTE domain to HEC1 (F). The prevention of MR binding to NUF2 lowers the overall affinity of MPS1 for the NDC80 complex and reduces the kinetochore residence time leading to relatively high turnover rates.

In humans, the localization of MPS1 to kinetochores relies on the microtubule-binding NDC80 complex through two interaction sites [19,21,39,94–97]. First, the N-terminal region of MPS1 containing the NTE and TPR domains and which harbours the main kinetochore-binding capacity directly binds to a region in the calponin homology domain of NDC80 complex subunit HEC1 [14,15] (figure 2E,F). The MPS1-interacting residues in HEC1 were mapped to a region close to the HEC1–microtubule interface [14]. Second, a conserved motif in the mostly uncharacterized middle region (MR; figure 1, box 1) of MPS1 interacts with the CH domain of NUF2, the obligatory binding partner of HEC1 [15] (figure 2E). Although important for SAC activity, this interaction contributes little to the overall protein levels of MPS1 at kinetochores [15,58]. However, inactive versions of MPS1 rely more heavily on the MR–NUF2 interaction [58], suggesting different interaction modes: an initial MR–NUF2 interaction that diminishes upon MPS1 activation, leaving the NTE–HEC1 interaction to act as the predominant localization mode throughout mitosis [58]. Although described as separate modes of interaction, there is no direct evidence against a model in which a single molecule of MPS1 can interact with the kinetochore via both interaction sites (figure 2E). Nevertheless, the mechanism behind the functional significance of the MR–NUF2 interaction remains to be determined: while it affects the SAC, it does not appear to impact the steady-state levels that active MPS1 can reach on kinetochores of nocodazole-treated cells [15,58]. Lastly, it cannot be excluded that MPS1 has additional binding partners on the kinetochore. However, if such interactions take place, they are likely of low affinity or affect a small pool of MPS1 and have so far never been observed.

## 6. Under control: regulators of the interaction of Mps1 with kinetochores

Kinetochore localization of human MPS1 depends on the activity of Aurora B, which promotes rapid activation of SAC signalling at the onset of mitosis [19,21]. The mechanism by which Aurora B impacts MPS1 localization is largely unknown. Aurora B stimulates MPS1–NDC80 interaction by releasing an inhibitory effect imposed on the NTE by MPS1's TPR domain [39] (figure 2A,C). No direct Aurora B phosphorylation sites have been reported on NTE or TPR, so whether Aurora B does so directly or indirectly remains unknown. Aurora B may also stimulate MPS1–NUF2 interactions: at least *in vitro*, Aurora B frees the MR peptide-binding site on NUF2 from occupation by the tail of HEC1 [15] (figure 2B,E). It will be of interest to examine if an Aurora B-insensitive (9A) version of HEC1 hampers efficient localization of inactive MPS1 to prophase kinetochores. Recently, a mitotic role was uncovered for the guanine nucleotide exchange factor, ARHGEF17 [98,99]. In mitosis, ARHGEF17 brings MPS1 to the kinetochore independently of its Rho GEF catalytic activity (figure 2D) [100]. ARHGEF17 forms a complex with inactive MPS1 to bring it to kinetochores, where the complex is disassembled after phosphorylation of ARHGEF17 by activated MPS1. There is no current insight into how the ARHGEF17–MPS1 interaction is established or regulated, but given the similar impact on MPS1 localization, it is possible that Aurora B is involved in this step (figure 2D).

MPS1 is heavily phosphorylated on its kinetochore localization module [74,76,101,102]. At least some sites are modified through autophosphorylation, but no thorough analysis of their individual contributions to MPS1 function has been done. It is likely that one or more phosphorylated sites contribute to localization, as phosphorylation of the NTE–TPR module stimulates binding to the NDC80 complex *in vitro* [14]. Other sites may impact on the release of MPS1 from kinetochores rather than initial binding [103] (see also §7 below). Yet more kinases can regulate MPS1: Cdk1, Chk2 and Map kinase all have at least one target residue on Mps1. Mutations of these residues interfered with the ability of Mps1 to either localize to kinetochores or to efficiently recruit downstream components of the SAC [104–108].

## 7. Let it go: a suggestion for a revised model of Mps1 release from kinetochores

Kinase-dead mutants of Mps1 and chemically inhibited Mps1 localize to higher levels on unattached kinetochores compared with their active counterparts [56,109–111]; this spurred a model in which Mps1 activity promotes its release. Mps1 molecules display rapid turnover on kinetochores which decreases significantly when the kinase is inactivated, at least partially explaining its local accumulation under those conditions [109,112]. The short residence time of Mps1 on kinetochores is important for normal mitotic progression, as preventing its release or perturbing the dynamics of autophosphorylation on NTE or MR causes metaphase delays or chromosome segregation errors, respectively [58,103,109].

The mitotic kinase Plk1 also contributes to the regulation of Mps1 release from kinetochores. Plk1 activity promotes Mps1 dissociation from kinetochores [113], and *in vitro* Plk1 can hit the same sites as Mps1 can, which is consistent with the fact that the optimal sequence of their phosphorylation targets is similar [33,113]. In cells, inhibition of Plk1 activity leads to elevated Mps1 kinetochore levels as a result of slower turnover, similar to Mps1 inhibition. Interestingly, inhibition of both kinases has an additive effect, indicating that not only the shared target sites but also the ones that are specific for each kinase contribute to Mps1 turnover. As Plk1 localizes to kinetochores before mitosis [114], it may have a particularly important role in potentiating Mps1 functionality at the transition to mitosis or in its early phases [113].

The current model for how Mps1 dissociates from kinetochores assumes that release is actively promoted by Mps1. There is, however, an alternative model in which the different turnover rates of active and inactive molecules reflect different modes of Mps1 interaction with the Ndc80 complex. As mentioned earlier, inactive MPS1 accumulates on kinetochores to higher levels than active MPS1. A recent study showed that the MR region contributes only to MPS1 kinetochore localization when the protein is inactive, but is dispensable for localization of active MPS1 (figure 2E) [58]. Deletion of the MR region did not, however, abolish all ability of inactive MPS1 to localize but appeared to abolish only the increase in localization seen when inactive MPS1 is compared to active MPS1 [58]. This suggests that while the NTE is predominantly responsible for the binding of active MPS1 to kinetochores, both the NTE and MR regions are involved in kinetochore binding of the inactive form (figure 2E). As shown recently, activation of MPS1 leads to extensive conformational changes in the protein [79] which could affect the availability of its kinetochore-binding regions. When inactive, both the NTE and MR regions are available, resulting in relatively high affinity of MPS1 for the NDC80 complex, and thus relatively long residence times on kinetochores and high steady-state levels (figure 2E). Activation of MPS1 subsequently causes it to adopt a different conformation [79] that potentially prevents the MR–NUF2 interaction, as recently suggested (figure 2F) [58]. The fully active MPS1 molecule now only interacts with the NDC80 complex via its NTE, lowering overall affinity for the NDC80 complex, and thereby decreasing residence time and levels at mitotic kinetochores (figure 2F). Under this model, therefore, MPS1 activity prevents the MR–NUF2 interaction from taking place by inducing conformational changes to the protein, rather than diminishing the interaction between the NTE and NDC80 by phosphorylating the NTE. The functional implications of such regulation are that the longer kinetochore residence times of inactive MPS1 molecules could facilitate more efficient *trans*-autophosphorylation and thus promote their activation. Once active, switching to a mode of higher turnover of MPS1 molecules on kinetochores is important for rapid dampening of the SAC signal once microtubule attachments are formed (discussed in more detail in §8).

## 8. Keep ‘em separated: blocking access of Mps1 to its kinetochore substrates

**Figure 3.**
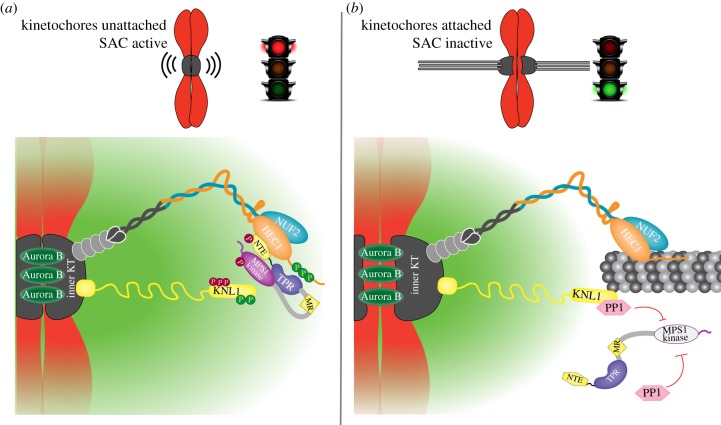
Mechanisms of removal of MPS1 activity from kinetochores. When kinetochores are unattached (*a*), Aurora B activity is high and helps recruit MPS1 to the NDC80 complex. From there, MPS1 phosphorylates its kinetochore substrates which eventually leads to SAC activity and mitotic arrest. After initial microtubule capture by kinetochores (*b*), MPS1 molecules no longer efficiently interact with NDC80 complexes that are bound by microtubules. Subsequent stabilization of end-on attachments and chromosome biorientation results in further displacement of the majority of MPS1 from kinetochores. Additionally, MPS1 molecules become inactivated by phosphatases whose activity increases in the cell and can be recruited to kinetochores due to the tension-dependent reduction in outer kinetochore Aurora B activity.

In order for anaphase to initiate, SAC silencing needs to occur once all chromosomes have formed stable attachments to microtubules of the mitotic spindle [115,116]. As Mps1 is the master regulator of SAC signalling in many different organisms, key to SAC silencing is to prevent it from phosphorylating its substrates. As expected, Mps1 levels on kinetochores are drastically reduced once stable end-on attachment to microtubules is achieved [14], and forced presence of Mps1 on metaphase kinetochores leads to a failure to silence the SAC [109]. A major mechanism for microtubule-dependent reductions in kinetochore MPS1 levels in humans is competition between MPS1 and microtubules for overlapping binding sites. The NTE binds close to the NDC80–microtubule interface (figure 3*a*), and microtubules compete with NTE–NDC80 interactions *in vitro* [14,15]. In addition, microtubules also prevent interactions between NUF2 and an MR peptide [15]. As MPS1 cycles dynamically on and off the kinetochore, it is probing the attachment status via its ability to re-bind. Once a microtubule has formed a stable end-on attachment, MPS1 is precluded from reaching its binding site on the kinetochore and thus phosphorylation of its substrates is prevented, leading to a discontinuation of SAC signalling (figure 3*b*). Another potential consequence of this is that MPS1 molecules, now unable to cluster on kinetochores, cannot be efficiently reactivated once they become inactive. MPS1's ability to re-bind may also depend on Aurora B activity, and metaphase tension states may thus further ensure that re-binding is prevented. Molecular insights into how Aurora B promotes MPS1 localization will determine whether this is the case.

Residual low levels of Mps1 remain on metaphase kinetochores [14,117], yet this pool is unable to elicit a SAC response. The reasons for this are not fully known, but several mechanisms can be responsible. First, in budding yeast, the mechanical tension exerted on kinetochores by stably bound microtubules results in the physical separation of Mps1 kinase molecules from their substrates, with the Dam1 ring complex acting as a further potential barrier between kinase and substrates [118]. This mechanism may not apply to human cells, as there is evidence not only for substantial cytoplasmic Mps1 activity but also for high Mps1 mobility, which is unlikely to be hampered by physical barriers to nearby substrates [51,119]. Second, protein phosphatase 1 (PP1) (and possibly PP2A) is necessary to switch off SAC signalling by reverting Mps1 substrate phosphorylation [71,120–122] and, at least in *Drosophila* cells, by directly inactivating the kinase [123]. Aurora B normally prevents PP1 recruitment to kinetochore by phosphorylating its kinetochore-docking RVxF motif in Knl1 [124,125]. A decrease in outer kinetochore Aurora B activity thus promotes PP1 recruitment due to RVxF dephosphorylation. High PP1 levels on bioriented kinetochores may have a relatively easy time inactivating the few residual molecules of Mps1 still localized there, as well as dephosphorylating Mps1 substrates (figure 3*b*). Cytoplasmic PP1 activity can further assist this process.

## 9. All together now: a temporal model for human MPS1 function in mitosis

Aside from a possible small pool of active MPS1 prior to mitosis, the majority of MPS1 molecules are inactive. When kinetochores are assembled, MPS1 is recruited to the NDC80 complexes via its NTE and MR regions (figure 2E). This causes kinetochore accumulation of inactive MPS1 molecules with relatively low turnover (figure 2E), leading to peak MPS1 kinetochore levels in prophase [21]. The long residence time facilitates *trans*-autophosphorylation, resulting in rapid activation of a substantial pool of MPS1. This pool is now proficient in eliciting a strong SAC signal around the time of nuclear envelope breakdown (figure 3*a*). Activation of MPS1 now causes the MR–NDC80C interaction to diminish, increasing its turnover rate and decreasing its steady-state kinetochore levels (figure 2F). This allows probing of the attachment state of chromosomes and facilitates MPS1 inactivation upon MT capture. When stable end-on kinetochore–microtubule attachments are formed, MPS1 is precluded from re-binding to those kinetochores, reducing the overall SAC signal in cells (figure 3*b*). As more and more kinetochores become attached, this signal is further dampened. In parallel, biorientation of chromosomes reduces Aurora B activity near kinetochores, promoting PP1 binding. PP1 then dephosphorylates Mps1 substrates and potentially directly inactivates any residual Mps1 molecules at or near kinetochores, essentially quenching the SAC signal emitted from these bioriented chromosomes (figure 3*b*). Relocalization of MPS1 to attached kinetochores at this stage is severely hampered by the microtubules, further ensuring that inactivated MPS1 can no longer be reactivated. When a sufficiently large pool of MPS1 is inactivated, all SAC activity is quenched and APC/C activity is initiated.

## 10. What else is there: outstanding questions

We have outlined current knowledge on how localization and activity of the chief executive officer of the SAC, the kinase Mps1, is regulated. Understanding its role in the protection of genome stability is far from complete, however, and several key lacunas need to be filled.

Though Mps1 activity peaks in mitosis, Mps1-dependent formation of MCC complexes already occurs in interphase. Very little is known about how and where this pool of Mps1 becomes activated. Answers will be greatly assisted by the development of a live biosensor assay, similar to those that clarified essential features of Aurora B activity dynamics [126]. Upon entry into mitosis, Mps1 recruitment to unattached kinetochores is necessary for the wave of activation that is needed to mount a full SAC response. Several questions as to how this is achieved are as yet unanswered. How does dimerization of Mps1 occur and how does it affect the activation dynamics of the kinase? What are the steps leading to full activation of the kinase, and what are the roles of specific NTE and MR modifications? The recent finding of a possible inactive ‘prone-to-autophosphorylate’ conformer of Mps1 involving the NTE implies an even more complex activation mechanism than currently envisioned [79]. How exactly are Aurora B and ARHGEF17 involved in these initial Mps1–kinetochore interactions (figure 2C,D)? Have all contributing factors been identified? Genetics and proteomics approaches have improved to such an extent that repeating several screens for SAC modifiers may yield novel players. Finally, non-kinetochore-bound Mps1 activity can generate an SAC signal and, in some cases, support a mitotic arrest [51,80,127]. Is there, however, a role for such non-kinetochore Mps1 activity in the maintenance of a mitotic arrest after a full initial kinetochore-dependent SAC response has been mounted?

Competition with microtubules ensures that Mps1 kinetochore levels become greatly reduced once stable attachments have been formed and this is a major SAC silencing mechanism. Once Mps1 is displaced from kinetochores, how exactly does inactivation take place and what are the dynamics of it? Nevertheless, a fraction of Mps1 remains bound to kinetochores even in the presence of stable attachments [14]. This begs the question of where this residual Mps1 is binding and how much reduction of Mps1 levels is sufficient to tip the balance in favour of the phosphatases and switch off SAC signalling. Although HEC1 tail dephosphorylation is crucial for the establishment of stable microtubule attachments, not all HEC1 molecules at a human kinetochore (approx. 240 [128]) are completely de-phosphorylated and not all HEC1 molecules are attached to microtubules when anaphase ensues [129]. Are the incompletely de-phosphorylated HEC1 molecules the ones that retain MPS1 due to a lower affinity for microtubules? Is there a gradual reduction in Mps1 levels that tracks ever increasing microtubule occupancy, or is there a more switch-like behaviour? Does SAC strength correlate with Mps1 kinetochore levels? Finally, how is removal of Mps1 from microtubule-bound kinetochores compatible with its role in error correction? Can error correction be maintained by, for example, less Mps1 activity than needed for the SAC, or are relevant error-correction and SAC substrates affected differently by the same reductions in Mps1 activity?

Much progress has been made in recent years with regard to understanding the principles that underlie faithful chromosome segregation and normal mitotic progression. The importance of attachment error correction and the SAC in these processes and the involvement of Mps1 are undisputed. Elucidating the finer points of Mps1 regulation in the coming years will provide crucial insights into how genomic stability is ensured.

## Data accessibility

This article has no additional data.
